# Targeting Palbociclib-Resistant Estrogen Receptor-Positive Breast Cancer Cells via Oncolytic Virotherapy

**DOI:** 10.3390/cancers11050684

**Published:** 2019-05-16

**Authors:** Nadiia Lypova, Lilibeth Lanceta, Alana Gipson, Stephanie Vega, Rodolfo Garza-Morales, Kelly M. McMasters, Jason Chesney, Jorge G. Gomez-Gutierrez, Yoannis Imbert-Fernandez

**Affiliations:** 1 Department of Medicine, School of Medicine, University of Louisville, Louisville, KY 40202, USA; nadiia.lypova@louisville.edu (N.L.); lilibeth.lanceta@louisville.edu (L.L.); s0vega01@louisville.edu (S.V.); jason.chesney@louisville.edu (J.C.); 2 The Hiram C. Polk Jr., MD, Department of Surgery, School of Medicine, University of Louisville, Louisville, KY 40202, USA; alana.gipson@louisville.edu (A.G.); rodolfo.garzamorales@louisville.edu (R.G.-M.); kelly.mcmasters@louisville.edu (K.M.M.); jgguti01@louisville.edu (J.G.G.-G.); 3 James Graham Brown Cancer Center, School of Medicine, University of Louisville, Louisville, KY 40202, USA

**Keywords:** palbociclib, estrogen receptor, oncolytic adenovirus, breast cancer, virotherapy, CDK4/6, CDK4/6 inhibitors, therapy resistance

## Abstract

While clinical responses to palbociclib have been promising, metastatic breast cancer remains incurable due to the development of resistance. We generated estrogen receptor-positive (ER+) and ER-negative (ER−) cell line models and determined their permissiveness and cellular responses to an oncolytic adenovirus (OAd) known as Ad5/3-delta24. Analysis of ER+ and ER− palbociclib-resistant cells revealed two clearly distinguishable responses to the OAd. While ER+ palbociclib-resistant cells displayed a hypersensitive phenotype to the effects of the OAd, ER− palbociclib-resistant cells showed a resistant phenotype to the OAd. Hypersensitivity to the OAd in ER+ palbociclib-resistant cells correlated with a decrease in type I interferon (IFN) signaling, an increase in viral entry receptor expression, and an increase in cyclin E expression. OAd resistance in ER− palbociclib-resistant cells correlated with an increase in type I IFN signaling and a marked decrease in viral entry receptor. Using the OAd as monotherapy caused significant cytotoxicity to both ER+ and ER− palbociclib-sensitive cell lines. However, the addition of palbociclib increased the oncolytic activity of the OAd only in ER+ palbociclib-sensitive cells. Our studies provide a mechanistic base for a novel anti-cancer regimen composed of an OAd in combination with palbociclib for the treatment of ER+ breast cancer.

## 1. Introduction

Breast cancer is the most common malignancy in women, and one of the three most common cancers worldwide [1]. Despite remarkable therapeutic advances, metastatic breast cancer remains an incurable, systemic disease—in 2019, an estimated 41,760 individuals will die of breast cancer [2]. Over 70% of all breast malignancies express the estrogen receptor (ER), thus many of these primary tumors benefit from endocrine therapies (ET) such as antiestrogens or aromatase inhibitors [3,4].

While endocrine treatment is an effective first-line therapy for ER+ breast cancer, its success is limited by either intrinsic or acquired resistance [5]. Cyclin D1 amplification has been shown to compromise the response to ET by promoting the activation of the cyclin-dependent kinases 4 and 6 (CDK4/6), leading to cell cycle progression, even in the absence of estrogen [6,7,8,9]. These observations prompted the development of the first-in-class, oral, small-molecule inhibitor of the catalytic domains of CDK4/6, palbociclib (PD-0332991, Ibrance) [10,11,12,13,14]. To date, three orally bioavailable CDK4/6 inhibitors (palbociclib, ribociclib, abemaciclib) have been FDA-approved for the treatment of advanced ER+ breast cancer [15]. CDK4/6 inhibitors such as palbociclib induce arrest in the G1 phase of the cell cycle in cancer cells and inhibit tumor growth in vivo [16]. Palbociclib is approved for use in combination with ET as a first-line treatment against ER+ advanced breast cancer. Recently, a number of preclinical studies have shown that CDK4/6 inhibitors also demonstrate activity against triple-negative breast cancer (TNBC), which lack the expression of ER, progesterone, and Her2 [17,18,19]. As a result, several clinical trials evaluating CDK4/6 inhibitors are currently underway (clinical trials.gov; NCT02978716, NCT03519178, NCT03805399, NCT03090165). Despite promising clinical outcomes, intrinsic or acquired resistance to CDK4/6 inhibitors has limited the success of these treatments; therefore, the development of various strategies to overcome this resistance is of great importance.

One of the most promising approaches for the treatment of malignant tumors is the use of oncolytic adenoviruses (OAds). OAds are genetically modified viruses that selectively replicate, spread, and induce oncolytic cell death in cancer cells without harming normal cells [20]. Preclinical and clinical studies indicate that the combination of OAds with other treatment modalities, such as radiotherapy and chemotherapeutic agents, increases the anticancer effects of OAds [21,22,23,24,25]. Some host factors that are key for the OAds to successfully complete infection and oncolysis are the cellular antiviral interferon (IFN) response and cell cycle machinery [26,27]. Specifically, previous studies have shown that heightened IFN signaling decreases the therapeutic efficacy of OAds [28,29]. Further, we and others have previously reported that cyclin E overexpression and the activation of CDK2 positively correlate with OAd replication and are required for the activity of an OAd [30,31]. Coincidentally, potential mechanisms of resistance to CDK4/6 inhibition include deregulation of the antiviral IFN response and aberrant cell cycle progression due to high levels of cyclin E and the activation of CDK2 [32,33,34]. Thus, we hypothesized that combining palbociclib with oncolytic virotherapy will be a promising approach for treating both palbociclib-sensitive and palbociclib-resistant breast cancer.

In the current study, we evaluated the ability of an OAd known as Ad5/3-delta24 to replicate and cause oncolysis on ER+ and ER− palbociclib-sensitive and palbociclib-resistant breast cancer cells. We also examined the roles of type I IFN antiviral response and cyclin E expression on breast cancer oncolytic virotherapy efficacy. The findings demonstrate that OAd replication and oncolysis was more efficient in palbociclib-resistant ER+ breast cancer cells due to their low antiviral IFN response and increased CDK2 activation. Further, we report that while an OAd exerted significant oncolytic activity in both ER+ and ER− palbociclib-sensitive breast cancer cells, the addition of palbociclib augmented the oncolytic activity only in ER+ breast cancer cells. Our data support the further investigation of OAds in combination with other CDK4/6 inhibitors in vivo to determine the potential of this strategy to prolong the response time and overcome resistance to palbociclib, which will ultimately impact the survival of ER+ breast cancer patients.

## 2. Results

### 2.1. Generation of ER+ and ER− Palbociclib-Resistant Breast Cancer Cell Models

**Figure 1:**
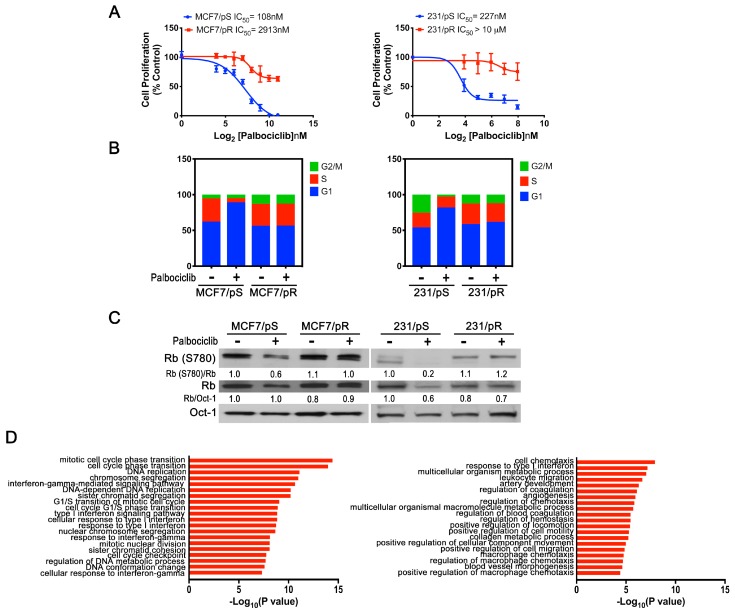
Characterization of ER+ and ER− palbociclib-resistant breast cancer cell lines. (**A**) ER+ MCF7 and ER− MDA-MB-231 cells (sensitive and resistant to palbociclib) were treated with the indicated concentrations of palbociclib for 72 h. Relative cell proliferation was determined by a FluoReporter assay. Percent inhibition of proliferation was calculated as a function of the number of cells compared to the control. Each data point represents the average of three independent experiments. (**B**) Cells were treated with either the vehicle (H_2_O) or palbociclib (500 nM) for 24 h followed by flow cytometry cell cycle analysis. The results from a representative experiment are shown. (**C**) Protein levels from nuclear extracts were evaluated by Western blot 24 h after treatment with 500 nM palbociclib. Oct-1 is shown as the loading control. Quantitative densitometry analysis is shown as the ratio of Rb(S780) to Rb and Rb to Oct-1 normalized to their respective control-treated pS cells. (**D**) Top 20 enriched Gene Ontology Biological (GO:BP) terms for differentially expressed genes (DEG) from MCF7/pR vs. MCF7/pS and 231/pR vs. 231/pS (*p*-value ≤ 0.05; *q*-value ≤ 0.05; |log2FC| ≥ 1).

Despite the initial success of anti-CDK4/6 agents such as palbociclib in ER+ breast cancers, drug resistance remains a major challenge for a significant subset of patients. We predicted that palbociclib-resistant tumors would acquire new “dependencies” that we could exploit to develop a new therapeutic strategy. To identify such vulnerabilities, we initially developed ER+ and ER− breast cancer cell line models of acquired resistance to palbociclib. We continuously exposed MCF7 (ER+) and MDA-MB-231 (ER−) cells to increasing doses of palbociclib (0.1–4 μM) over a span of 6 months and monitored cell growth. We confirmed that the established palbociclib-resistant MCF7 (MCF7/pR) and MDA-MB-231 (231/pR) cells display a significant increase in half maximal growth inhibitory concentration (IC_50_) compared to the parental palbociclib-sensitive MCF7 (MCF7/pS) and MDA-MB-231 (231/pS) cells [IC_50_: MCF7/pS = 108 ± 13.15 nM; MCF7/pR = 2913 ± 790 nM and 231/pS = 227 ± 59.41 nM; 231/pR = 18,081 ± 3696 nM] (Figure 1A). We next performed cell cycle analysis and, as expected, we observed a marked G1 arrest in MCF7/pS and 231/pS cells exposed to palbociclib [14]. In contrast, palbociclib treatment had no effect on cell cycle distribution in MCF7/pR or 231/pR cells (Figure 1B). To determine whether resistance to palbociclib was due to an impairment of the cyclin D1–CDK4/6–Rb axis, we examined the effect of palbociclib on Rb phosphorylation at serine 780 (RbS780). Palbociclib treatment led to a decrease in Rb phosphorylation only in MCF7/pS and 231/pS cells (Figure 1C). Taken together, these data demonstrate that MCF7/pR and 231/pR cells are resistant to inhibition by palbociclib and maintain persistent Rb phosphorylation in the presence of palbociclib.

To identify differentially expressed genes and pathways involved in the development of resistance to palbociclib, we performed gene expression (GE) analysis of palbociclib-sensitive and palbociclib-resistant ER+ MCF7 and ER− MDA-MB-231 breast cancer cells. Analysis of ER+ MCF7/pR compared to MCF7/pS cells showed differentially regulated genes with Gene Ontology Biological Processes (GO:BP) terms related to “cell cycle transition”, “mitosis” and “DNA replication” (Figure 1D). Interestingly, we also observed that GO:BP processes such as “response to type I IFN”, “type I IFN-signaling pathway” and “response to IFN-gamma” were enriched in ER+ MCF7/pR cells compared to MCF7/pS cells. Notably, while GE analysis of ER− 231/pR compared to 231/pS revealed an enrichment of GO:BP terms related to “chemotaxis”, “cell migration” and “metabolic process”, it also showed “response to type I IFN” as seen in ER+ MCF7/pR cells.

### 2.2. Acquired Resistance to Palbociclib Promotes Sensitivity to OAdmCherry Only in ER+ MCF7 Breast Cancer Cells

**Figure 2:**
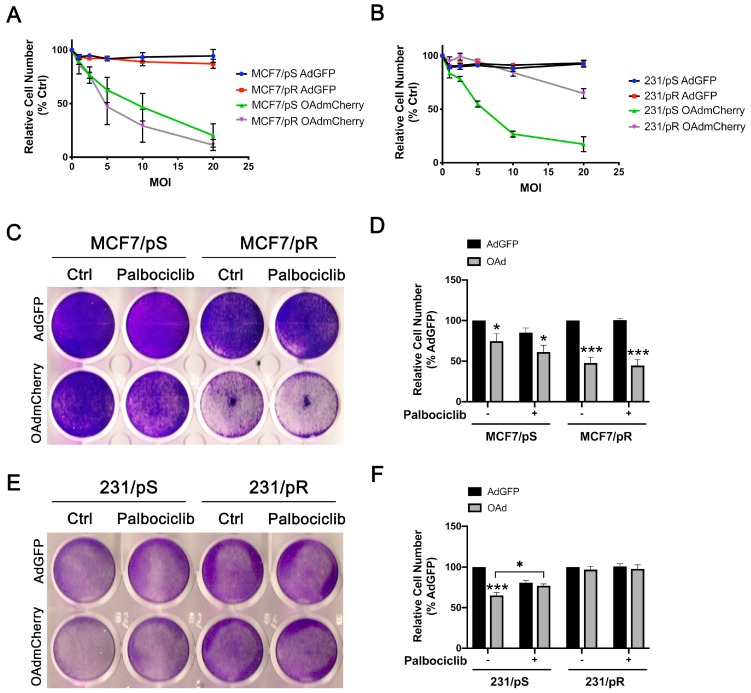
ER+ and ER− breast cancer cell lines exhibit different cytotoxicity in response to the Ad5/3-delta24 modified to express the mCherry protein (OAdmCherry). (**A**,**B**) Cells were infected with adenovirus expressing green fluorescent protein (AdGFP) or OAdmCherry at the indicated multiplicity of infection (MOI) for 48 h. Cell cytotoxicity was assessed by crystal violet staining. OAd-mediated cytotoxicity was calculated by measuring the absorbance of solubilized dye at 590 nm and is expressed as percent (%) cell number relative to AdGFP vehicle-treated cells. The results shown are from one experiment. Three technical replicates were used to measure dye absorbance. (**C**,**E**) Cells were infected with a replication-deficient adenovirus expressing the green fluorescent protein (AdGFP) or the Ad5/3-delta24 modified to express the mCherry protein (OAdmCherry) at a multiplicity of infection (MOI) of five alone or in combination with palbociclib (500 nM) for 48 h. Cell survival was assessed by crystal violet staining. Representative staining for each cell line of three independent experiments performed is shown. (**D**,**F**) OAd-mediated cytotoxicity was calculated by measuring the absorbance of solubilized dye at 590 nm and is expressed as percent (%) cell number relative to their respective AdGFP vehicle-treated control. Error bars, ±SEM of four independent experiments. * *p* < 0.05; *** *p* < 0.001; compared to AdGFP unless otherwise indicated.

The ability of oncolytic viruses to infect and replicate within cells is highly dependent on the type I IFN pathway, the major cellular antiviral response [28]. Given that our GE analysis identified changes in “response to type I IFN” in palbociclib-resistant MCF7 and MDA-MB-231 cells, we sought to determine their susceptibility to an OAd known as Ad5/3-delta24 that has been modified to express mCherry (OAdmCherry) [35]. First, we determined the optimal dose of OAdmCherry able to induce cytotoxicity in MCF7/pS, MCF7/pR, 231/pS and 231/pR breast cancer cells in the absence or presence of palbociclib. Briefly, cells were infected with either OAdmCherry or a replication-deficient adenovirus expressing green fluorescent protein (AdGFP) followed by crystal violet staining 48 h post-infection to determine virus-induced cytotoxicity. Crystal violet staining revealed a dose-dependent increase in cytotoxicity in all OAdmCherry-infected cells. In contrast, AdGFP did not cause cytotoxicity in any of the cell lines tested, even at the highest multiplicity of infection (MOI) concentration (Figure 2A,B). In subsequent experiments, we used a sublethal dose of 5 MOI.

To study the combinatorial efficacy of OAdmCherry and palbociclib to promote tumor cell lysis, we measured OAdmCherry-mediated cytotoxicity by crystal violet staining in MCF7/pS, MCF7/pR, 231/pS and 231/pR breast cancer cells in the absence or presence of palbociclib. We found that infection with OAdmCherry resulted in greater cytotoxicity in MCF7/pR cells compared to MCF7/pS cells and that the addition of palbociclib increased the OAdmCherry-driven cytotoxicity in MCF/7pS (Figure 2C,D). In contrast, OAdmCherry infection of ER− MDA-MB-231 cells led to significant oncolytic activity only in 231/pS cells (Figure 2E,F). Notably, the addition of palbociclib or the development of resistance to palbociclib in ER− MDA-MB-231 breast cancer cells inhibited the oncolytic activity of OAdmCherry. To control for unanticipated cytotoxic effects of the adenovirus vector, we infected cells with AdGFP alone or in combination with palbociclib. We found that AdGFP alone caused no cytotoxicity in any of the cell lines tested, while the addition of palbociclib resulted in a 20% decrease in cell number in palbociclib-sensitive cells. Overall, our studies indicate that palbociclib exposure and acquired resistance to palbociclib increases the oncolytic activity of OAdmCherry only in ER+ MCF7 breast cancer cells.

### 2.3. Palbociclib Enhances OAdmCherry Replication and Oncolytic Properties Only in ER+ MCF7 Breast Cancer Cells

**Figure 3:**
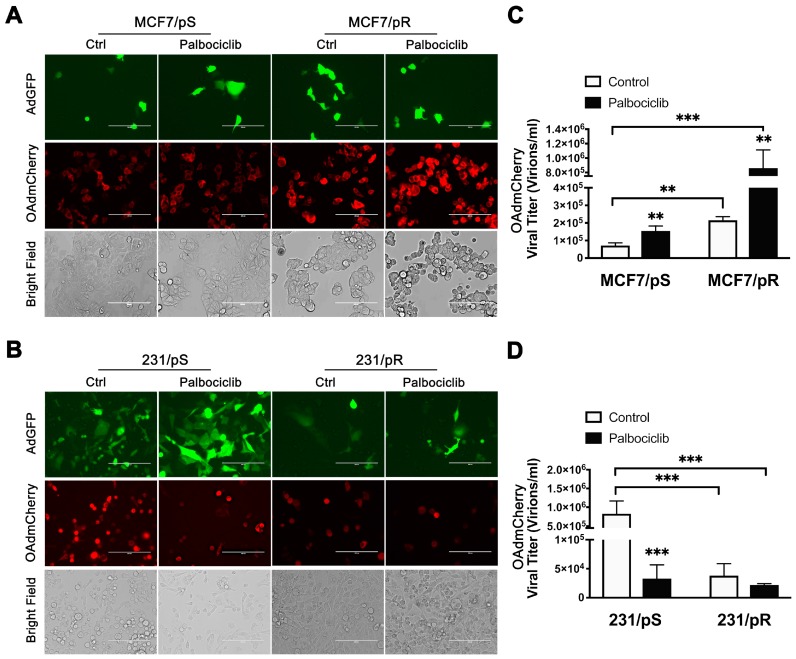
OAdmCherry replication in ER+ and ER− breast cancer cell lines. Cells were infected with AdGFP or OAdmCherry at a multiplicity of infection (MOI) concentration of five alone or in combination with palbociclib (500 nM) for 48 h. (**A**,**B**) Expression of GFP and mCherry was evaluated by fluorescence microscopy. Bright field images illustrate cytopathic effect (CPE). Scale: 200 µm. Images are representative of three independent experiments. (**C**,**D**) Viral titers were calculated from collected supernatants containing infectious viral particles released to the media. Error bars, ±SEM of three independent experiments. ** *p* < 0.01; *** *p* < 0.001; compared to the control unless otherwise indicated.

We used fluorescence microscopy to measure mCherry expression as a surrogate for OAdmCherry infectivity and replication efficiency. The expression of mCherry was significantly higher in MCF7/pR cells compared to MCF7/pS cells, indicating higher virus infectivity in palbociclib-resistant MCF7 cells. Moreover, the addition of palbociclib resulted in an increase in mCherry expression in both MCF7/pS and MCF7/pR cells (Figure 3A). The oncolytic property of OAdmCherry is best illustrated by the cytopathic effect (CPE). The CPE is defined as degenerative changes in cell morphology such as cell rounding and loss of cell adhesion associated with the replication of the OAdmCherry and resulting cancer cell damage. We found that OAdmCherry induced greater cell rounding (indicative of CPE) in MCF7/pR cells compared to MCF7/pS cells, and that the addition of palbociclib increased the CPE in both MCF7/pS and MCF7/pR cells (Figure 3A, bright field panel). We also examined ER-MDA-MB-231 cells after OAdmCherry infection and found significant mCherry expression in 231/pS control treated cells which was attenuated by the addition of palbociclib (Figure 3B). Moreover, bright field images showed greater CPE in 231/pS control-treated cells compared to palbociclib-treated 231/pS cells (Figure 3B, bright field panel). Further, the expression of GFP and mCherry along with CPE were significantly reduced in 231/pR cells compared to 231/pS cells.

To interrogate the ability of the virus to spread to neighboring cells, we calculated the release of OAdmCherry infectious particles to the media. We observed that MCF7/pR cells displayed increased viral particle production compared with MCF7/pS cells (Figure 3C). In addition, palbociclib exposure led to an increase in viral particle production in both MCF7/pS and MCF7/pR cells. Analysis of ER− cells revealed that virus titers were significantly higher in 231/pS control-treated cells compared to those treated with palbociclib (Figure 3D). Consistent with the low mCherry expression, 231/pR cells exhibited low OAdmCherry titers in the presence and absence of palbociclib. These results agree with our initial observation that 231/pR cells are resistant against the oncolytic activity of OAdmCherry.

Taken together, these data indicate that ER+ and ER− breast cancer cell models of acquired resistance to palbociclib have different permissiveness to OAdmCherry. While resistance to palbociclib increases the efficacy of OAdmCherry in ER+ MCF7 cells, it renders ER−MDA-MB-231 resistant to the oncolytic effects of OAdmCherry. Furthermore, combined palbociclib + OAdmCherry promotes replication, oncolysis and spread of OAdmCherry in ER+ MCF7 cells that are either sensitive or resistant to palbociclib. In contrast, palbociclib inhibits OAdmCherry replication, oncolysis and spread in ER− palbociclib-sensitive MDA-MB-231 cells.

### 2.4. Coxsackie-Adenovirus Receptor (CAR) Expression Correlates with Replication and Oncolytic Potential of OAdmCherry in Palbociclib-Resistant Cells

**Figure 4:**
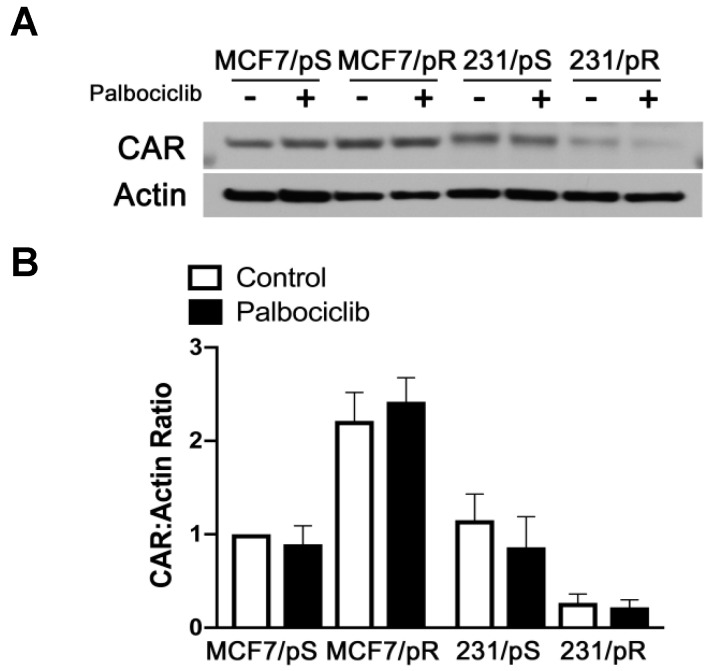
Coxsackie-adenovirus receptor (CAR) expression correlates with sensitivity to OAdmCherry. (**A**) Cells were treated with either the vehicle (H_2_O) or palbociclib (500 nM), harvested after 24 h and immunoblotted with the indicated antibodies. (**B**) Quantitative densitometry analysis is shown as the ratio of CAR to actin protein normalized to MCF7/pS vehicle-treated cells ± SEM from three independent experiments.

The inability of OAdmCherry to infect and/or replicate in 231/pR cells prompted us to examine the expression of the primary receptor for Ad5 known as CAR in all the cell lines. Immunoblot analysis revealed a marked decrease in CAR expression in 231/pR cells relative to 231/pS cells (Figure 4). We also noted a small decrease in CAR levels in 231/pS cells treated with palbociclib. Conversely, MCF7/pR cells showed a 2-fold increase in CAR expression compared to MCF7/pS cells. These results correlated with the level of sensitivity to OAdmCherry, where low-CAR-expressing 231/pR showed near complete resistance to OAdmCherry, while high-CAR-expressing MCF7/pR cells were hypersensitive to OAdmCherry.

### 2.5. Evaluation of Type I IFN Response in ER+ and ER− Palbociclib-Resistant Breast Cancer Cells

**Figure 5:**
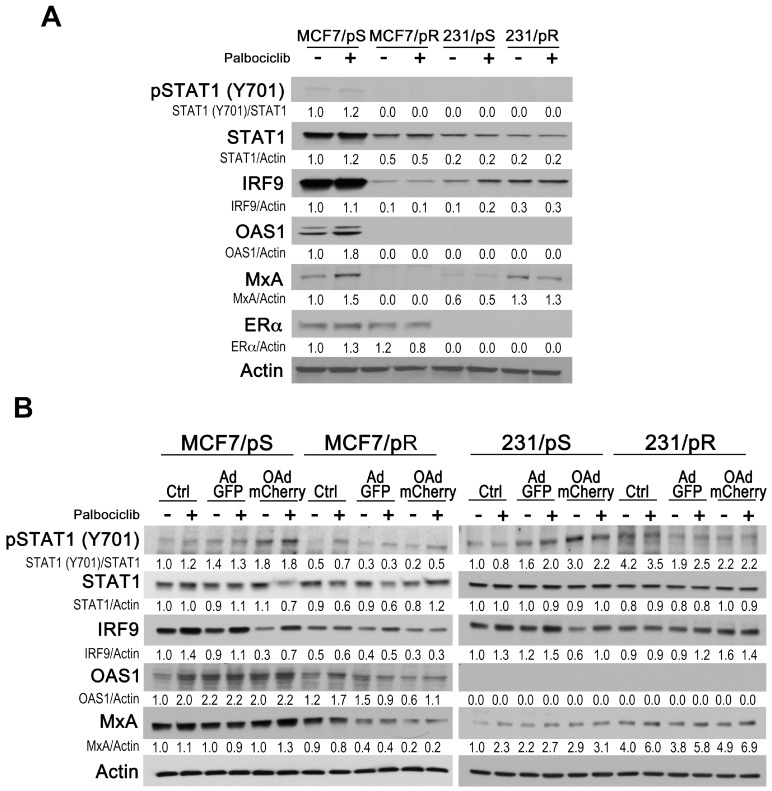
Type I IFN response in ER+ and ER− breast cancer cells. (**A**) Cells were treated with either the vehicle (H_2_O) or palbociclib (500 nM), harvested after 24 h, and immunoblotted with the indicated antibodies. (**B**) Cells were either left uninfected (Ctrl) or infected with AdGFP or OAdmCherry at a multiplicity of infection (MOI) concentration of five alone or in combination with 500 nM palbociclib for 24 h. Type I IFN signaling was assessed by immunoblotting with the indicated antibodies. Quantitative densitometry analysis is shown relative to loading control normalized to palbociclib-sensitive control-treated cells.

We hypothesized that differential sensitivity to OAdmCherry between ER+ MCF7 and ER− MDA-MB-231 cells that are either sensitive or resistant to palbociclib may also be due to differences in their antiviral type I IFN response. Upon viral stimulation, canonical IFN signaling results in the activation of Janus kinase 1 and/or tyrosine kinase 2 (JAK1/TYK2), which in turn, phosphorylates signal transducer and activator of transcription 1 (STAT1) and STAT2 [36]. Following phosphorylation, STAT1 binds IFN-regulatory factor 9 (IRF9) and forms the interferon-stimulated gene factor 3 complex (ISGF3) to induce the expression of hundreds of IFN-stimulatory genes (ISGs) with antiviral functions [37]. Initially, we evaluated the expression of IFN-signaling proteins (STAT1 (Y701), STAT1, STAT2, IRF9) and IFN-stimulated proteins that induce an antiviral state (OAS1 and MxA) in MCF7/pS, MCF7/pR, 231/pS and 231/pR breast cancer cells in the absence or presence of palbociclib. We found that STAT1 (Y701) and STAT1 levels were significantly reduced in MCF7/pR, 231/pS and 231/pR cells compared to MCF7/pS (Figure 5A). STAT2 expression was not detected in any of the cell lines tested. We observed that acquired resistance to palbociclib in ER+ MCF7 cells associated with a decrease in IRF9 expression. In contrast, increased IRF9 levels correlated with resistance to palbociclib in ER− MDA-MB-231 cells. The IFN-mediated antiviral response relies on MxA and OAS1 effector proteins to block viral transcription and degrade viral RNA, respectively [38]. Next, we examined MxA and OAS1 protein levels in the presence or absence of palbociclib in all cell lines. We found that OAS1 protein was constitutively expressed only in MCF7/pS cells, and that palbociclib exposure led to an increase in OAS1 expression. We observed that MxA levels were markedly suppressed in MCF7/pR relative to MCF7/pS cells. Conversely, 231/pR cells displayed increased MxA levels compared to 231/pS cells. In addition to testing type I IFN signaling, we also determined ERα levels and confirmed the expression of ERα in MCF7 cells but not in MDA-MB-231 cells. Taken together, our findings indicate that whereas resistance to palbociclib correlates with decreased type I IFN signaling and downstream effectors in ER+ MCF7 cells, it associates with increased type I IFN signaling in ER− MDA-MB-231 cells.

We next asked whether permissiveness to OAdmCherry in MCF7 and MDA-MB-231 cells that are either sensitive or resistant to palbociclib would correlate with type I IFN signaling. To test this, we treated cells with palbociclib followed by either no infection (Crtl) or infection with AdGFP or OAdmCherry prior to measuring type I IFN stimulation. We found that OAdmCherry infection induced type I IFN response as shown by the increase in STAT1 phosphorylation compared to uninfected control and AdGFP infected in MCF7/pS cells but not in MCF7/pR cells (Figure 5B). Similarly, infection with either AdGFP or OAdmCherry induced STAT1 phosphorylation in 231/pS cells but not in 231/pR cells. As part of the ISGF3 complex, IRF9 levels in response to STAT1 activation will determine the cellular antiviral state. We examined IRF9 expression following OAdmCherry infection and found a significant drop in IRF9 levels in MCF7/pS and 231/pS cells in the absence of palbociclib. These results are consistent with previous reports showing that adenovirus E1A protein decreases IRF9 levels thereby blocking the IFN-driven antiviral response [39]. Notably, palbociclib exposure blocked the ability of the OAdmCherry to decrease IRF9 levels in both MCF7/pS and 231/pS cells. Consistent with our initial observations, MCF7/pR cells displayed low levels of endogenous IRF9 compared to MCF7/pS cells, which were reduced further after OAdmCherry infection (Figure 5B). Analysis of 231/pR cells revealed that OAdmCherry infection in the presence or absence of palbociclib results in a small increase in IRF9 expression. Next, we measured the expression of OAS1 and MxA following OAdmCherry infection. We noted a minor increase in OAS1 and MxA levels in MCF7/pS cells indicating that OAdmCherry infection induces a type I IFN antiviral response. Likewise, 231/pS cells exhibited an increase in MxA expression in response to OAdmCherry infection, whereas OAS1 was not detectable in these cells, even after OAdmCherry infection. Analysis of palbociclib-resistant cells revealed a decrease in basal OAS1 and MxA levels in OAdmCherry-infected MCF7/pR cells, suggesting that the antiviral response is impaired in these cells. However, OAdmCherry infection of 231/pR cells had no significant effect on the already elevated MxA levels, indicating that 231/pR cells have a constitutively heightened antiviral state. Collectively, these studies indicate that acquired resistance to palbociclib induces a low antiviral state in ER+ MCF7 cells, suggesting that permissiveness to OAdmCherry is due to their decreased antiviral capacity [28]. Inversely, ER− 231/pR cells display an increase in antiviral capacity, which is consistent with their OAdmCherry-resistant phenotype.

### 2.6. Downregulation of IRF9 Increases OAdmCherry Replication and Oncolysis in Palbociclib-Sensitive Breast Cancer Cells

**Figure 6:**
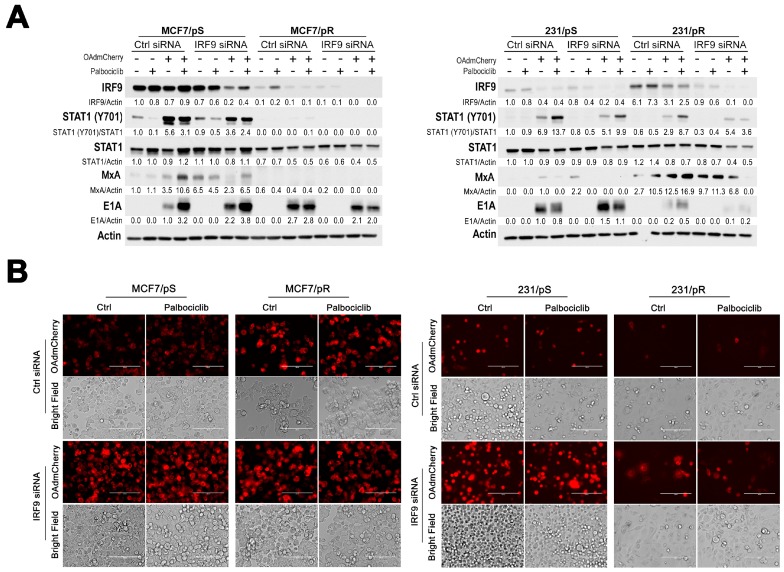

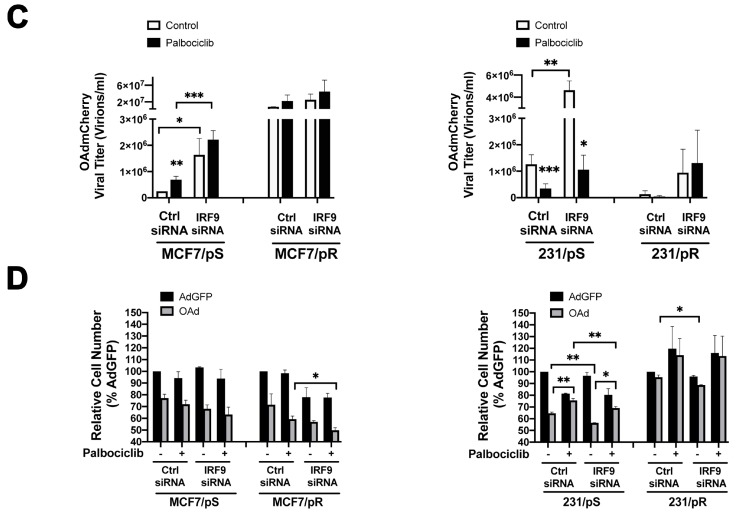
Regulation of viral replication and oncolysis by IFN-regulatory factor 9 (IRF9). Cells were transfected with either control or IRF9-specific siRNA #2 followed by infection with AdGFP or OAdmCherry at a multiplicity of infection (MOI) concentration of five alone or in combination with 500 nM palbociclib for 48 h. (**A**) Type I IFN signaling and expression of E1A viral protein was assessed by immunoblotting with the indicated antibodies. Quantitative densitometry analysis is shown relative to loading control normalized to palbociclib-sensitive control-treated cells (except for MxA in MDA-MB-231 cells and E1A blots where samples were normalized to OAdmCherry Ctrl). (**B**) mCherry expression was evaluated by fluorescence microscopy. Bright field images illustrate cytopathic effect (CPE). Scale: 200 µm. Images are representative of three independent experiments. (**C**) Cell lysates were harvested and combined with collected supernatants to determine OAdmCherry viral titers. (**D**) Cell number was assessed by crystal violet staining and calculated by measuring the absorbance of solubilized dye at 590 nm. Results are expressed as percent (%) of AdGFP vehicle-treated cells. Error bars, ±SEM of three independent experiments. * *p* < 0.05; ** *p* < 0.01; *** *p* < 0.001; compared to the control unless otherwise indicated.

Previous studies have indicated that upregulation of the IFN-regulated MxA gene contributes to resistance to oncolytic therapies [26,28]. To confirm that type I IFN signaling regulates sensitivity to OAdmCherry either alone or in combination with palbociclib, we performed siRNA experiments against IRF9 in palbociclib-sensitive and palbociclib-resistant MCF7 and MDA-MB-231 cells. We tested two IRF9-specific siRNAs and determined that their ability to knockdown IRF9 was comparable (Figure S1). Initially, we confirmed the decrease in IRF9 and MxA levels upon IRF9 siRNA transfection in all cell lines (Figure 6A). Additionally, we examined the effects of IRF9 siRNA on viral replication by measuring viral E1A protein expression. We found that IRF9 silencing led to a marked increase in E1A expression in control-treated MCF7/pS and 231/pS cells, while the addition of palbociclib resulted in a moderate increase in E1A levels in both cell lines. These results indicate that IRF9 silencing increases viral replication in MCF7/pS and 231/pS cells. Fluorescence microscopy showed that IRF9 silencing significantly increases mCherry expression and CPE in MCF7/pS, MCF7/pR and 231/pS in the presence and absence of palbociclib compared to their respective control siRNA (Figure 6B). However, only a minor increase in mCherry expression and CPE was noted in 231/pR cells upon IRF9 silencing, likely due to the low OAdmCherry transduction efficiency given their low CAR expression (Figure 4). Interestingly, palbociclib exposure of IRF9 siRNA transfected 231/pS cells decreased mCherry expression and CPE compared to the untreated IRF9-transfected cells, indicating that palbociclib inhibits OAdmCherry replication in 231/pS cells by an IRF9-independent mechanism. To further evaluate the effect of IRF9 silencing on OAd replication, we measured viral titers upon combined IRF9 siRNA and OAdmCherry infection. We found that siRNA targeting of IRF9 resulted in a significant increase in OAdmCherry titers in MCF7/pS cells in the presence or absence of palbociclib (Figure 6C). Similarly, IRF9 siRNA significantly increased OAdmCherry titers in control-treated 231/pS cells; however, palbociclib treatment blocked the increase in OAdmCherry replication, further confirming that the inhibitory effect of palbociclib on OAdmCherry replication in 231/pS cells is IRF9-independent. In MCF7/pR cells, we observed no significant enhancement in OAdmCherry titers after IRF9 silencing presumably due to the already low IRF9 levels. Likewise, IRF9 siRNA had no effect on OAdmCherry replication in 231/pR cells, which we attribute to the low transduction efficiency of OAdmCherry. Next, we examined the effect of IRF9 silencing on OAdmCherry-driven cytotoxicity and found that IRF9 knockdown increases the susceptibility of MCF7/pS and MCF7/pR cells to the oncolytic properties of the virus (Figure 6D). These results were consistent with the noted increase in E1A, mCherry expression, CPE, and viral titers in response to IRF9 siRNA. Likewise, IRF9 knockdown in 231/pS control-treated cells lead to a significant increase in virus-induced cytotoxicity compared to control siRNA, which was inhibited by the addition of palbociclib. Analysis of 231/pR cells upon IRF9 knockdown revealed a modest increase in OAdmCherry-driven cytotoxicity in palbociclib-untreated cells and this effect was inhibited by palbociclib. Taken together, these results indicate that targeting type I IFN via IRF9 silencing enhances the replication efficiency and oncolytic properties of OAdmCherry in palbociclib-sensitive MCF7 and MDA-MB-231 cells, thus confirming the role of the type I IFN pathway in promoting resistance to OAdmCherry. Furthermore, our studies suggest that increased permissiveness to OAdmCherry in MCF7/pR cells is, in part, due to IRF9 downregulation. Interestingly, IRF9 does not contribute to OAdmCherry resistance in palbociclib-treated 231/pS.

### 2.7. Cyclin E Knockdown Blocks Virus Replication Only in MCF7/pS Cells

**Figure 7:**
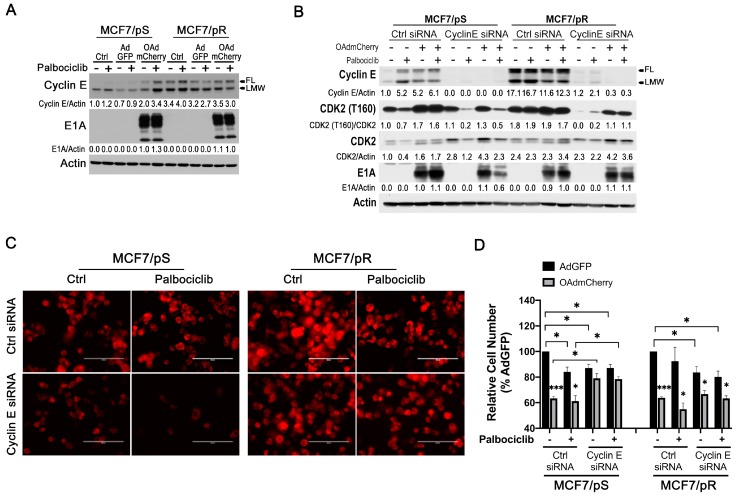
Cyclin E knockdown inhibits virus replication only in MCF7/pS cells. (**A**) Cells were either left uninfected (Ctrl) or infected with AdGFP or OAdmCherry at a multiplicity of infection (MOI) concentration of five alone or in combination with 500 nM palbociclib for 24 h. Cyclin E and viral E1A expression was assessed by immunoblotting. (**B**) Cells were transfected with either control or a pool of cyclin E-specific siRNAs followed by AdGFP or OAdmCherry at a MOI concentration of five alone or in combination with 500 nM palbociclib for 24 h. Cyclin E expression and downstream signaling as well as expression of E1A viral protein was assessed by immunoblotting. Quantitative densitometry analysis is shown relative to loading control normalized to palbociclib-sensitive control-treated cells (except for E1A blots where samples were normalized to OAdmCherry Ctrl). (**C**) mCherry expression was evaluated by fluorescence microscopy. Scale: 200 µm. (**D**) Cell number was assessed by crystal violet staining and calculated by measuring the absorbance of solubilized dye at 590 nm. Results are expressed as percent (%) of AdGFP vehicle-treated cells. Error bars, ±SEM of three independent experiments. * *p* < 0.05; *** *p* < 0.001; compared to control AdGFP unless otherwise indicated.

Preclinical and clinical research have shown that cyclin E overexpression and the aberrant activation of CDK2 is associated with acquired resistance to palbociclib [32,40]. Previous reports indicate that viral E1A protein increases cyclin E expression, which in turn, promotes the activation of CDK2 via its phosphorylation at the threonine 160 site [31]. Notably, this cyclin E induction and the subsequent activation of CDK2 is required for the replication and oncolytic activity of OAdmCherry [30]. Thus, we hypothesized that the increase in permissiveness for OAdmCherry seen in MCF7/pR cells would also be due to cyclin E overexpression. We measured cyclin E levels in MCF7/pS and MCF7/pR cells treated with palbociclib either alone or in combination with OAdmCherry for 24 h. It has been previously shown that cleavage of cyclin E generates a low-molecular weight (LMW) form of cyclin E, which is uniquely oncogenic and mediates resistance to palbociclib [40]. We found that MCF7/pR cells have a marked increase in both full-length (FL) and LMW forms of cyclin E compared to MCF7/pS cells (Figure 7A). We also observed an increase in the FL and LMW forms of cyclin E in OAdmCherry-infected MCF7/pS cells in the presence of palbociclib. Next, we sought to evaluate the role of cyclin E on OAdmCherry replication and OAdmCherry-mediated CPE in both MCF7/pS and MCF7/pR cells. To test this, we transfected cells with a pool of cyclin E-specific siRNAs followed by OAdmCherry infection cells alone or in combination with palbociclib. First, we confirmed downregulation of cyclin E and examined downstream T160 phosphorylation of CDK2 after OAdmCherry infection in cyclin E siRNA-transfected cells (Figure 7B). Consistent with previous studies, we found that infection with OAdmCherry significantly increases phospho-CDK2 levels in MCF7/pS cells, and that palbociclib had no effect on this OAdmCherry-driven increase in phospho-CDK2. In line with the high cyclin E expression seen in MCF7/pR cells, basal phospho-CDK2 levels were markedly increased relative to MCF7/pS cells, and OAdmCherry infection resulted only in a minor increase in phospho-CDK2. Cyclin E siRNA studies showed that, in the absence of cyclin E, OAdmCherry infection failed to induce CDK2 phosphorylation in MCF7/pS cells, and that this effect was further increased by palbociclib. In contrast, cyclin E siRNA in OAdmCherry-infected MCF7/pR cells resulted in a small decrease in phospho-CDK2 and palbociclib had no further effect. To determine the requirement of cyclin E for OAdmCherry replication, we measured E1A expression following cyclin E siRNA transfection. We found that cyclin E silencing led to a significant decrease in E1A expression levels only in MCF7/pS cells.

Analysis of mCherry expression showed that MCF7/pS cells transfected with cyclin E siRNA display reduced mCherry expression relative to control siRNA, and that this effect was greatly increased by palbociclib (Figure 7C). In contrast, cyclin E siRNA did not significantly affect mCherry expression in OAdmCherry infected-MCF7/pR cells with or without palbocilib, presumably due to the constitutive activation of CDK2 (Figure 7C). Finally, we examined the effect of cyclin E silencing on OAdmCherry-driven cytotoxicity and found that cyclin E knockdown significantly decreases the susceptibility of MCF7/pS cells to the oncolytic properties of OAdmCherry, and that the addition of palbociclib almost completely blocked the cytotoxic effect of OAdmCherry (Figure 7D). Interestingly, while cyclin E expression is required for the survival of MCF7/pR cells, cyclin E siRNA had no significant effect on OAdmCherry-driven cytotoxicity in the presence or absence of palbociclib in MCF7/pR cells. Together, these results indicate that cyclin E is required to maintain phospho-CDK2 levels in MCF7/pS cells, which in turn, facilitates OAdmCherry replication and oncolysis. In marked contrast, cyclin E expression is not required for OAdmCherry replication and oncolysis in MCF7/pR cells due to the constitutive activation of CDK2.

## 3. Discussion

In this study, we described the development of ER+ and ER− breast cancer cell lines with acquired resistance to palbociclib and evaluated their permissiveness to oncolytic virotherapy. We demonstrated that OAdmCherry used as monotherapy or in combination with palbociclib, causes significant cytotoxicities in both ER+ palbociclib-sensitive and palbociclib-resistant MCF7 breast cancer cells. Notably, the response to OAdmCherry was significantly increased in ER+ palbociclib-resistant MCF7 cells relative to the palbociclib-sensitive parental cells. On the contrary, palbociclib-resistant ER− MDA-MB-231 breast cancer cells showed minimal cytotoxicity in response to OAdmCherry either alone or in combination with palbociclib. Additionally, we showed that while palbociclib-sensitive MDA-MB-231 cells were susceptible to the cytotoxic effect of OAdmCherry, the addition of palbociclib blocked the virus replication and oncolytic activity.

Selective inhibition of CDK4/6 is now standard-of-care therapy for ER+ breast cancer, with concerted efforts underway to extend this therapy to other breast cancer subtypes, including TNBC. Three selective CDK4/6 inhibitors (palbociclib, abemabiclib, ribociclib) are currently FDA-approved based on their proven ability to increase progression-free survival in ER+ breast cancer patients [41,42,43,44]. While clinical responses to the CDK4/6 inhibitors are initially favorable, resistance almost invariably arises [45]. Combined with the fact that CDK4/6 inhibitors are not particularly effective as single agents, the need for combination therapies tailored to increase their efficacy and overcome resistance is increasingly evident. Oncolytic virotherapy is an emerging modality of tumor-targeting agents with proven safety against advanced cancers refractory to other therapies. While talimogene laherparepvec (T-VEC, a modified herpes simplex virus type 1) is the only oncolytic virus (OV) approved for treatment in the United States, an increasing number of OVs are in preclinical development or clinical trials [46,47]. Although OVs are already showing clinical potential in several solid tumors including breast cancer, their therapeutic efficacy is limited by inefficient replication [48,49]. Rational combination therapies aimed to maximize the efficiency of OVs are currently being tested, but identifying which combinations will be the most effective in patients remains challenging [49,50,51].

**Figure 8:**
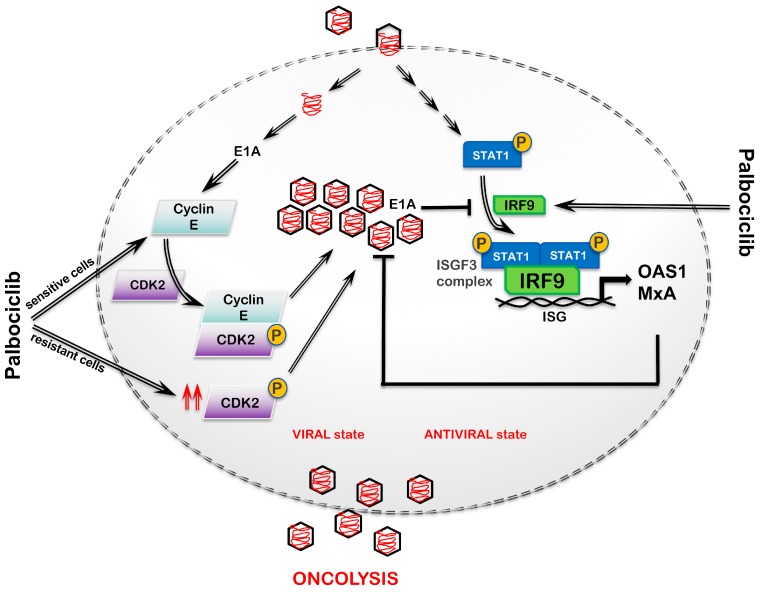
Model of interaction between an oncolytic virus and the host type I IFN response and cyclin expression. Host pattern recognition receptors (PRR) will recognize intracellular viral particles and initiate a type I IFN response. Upon viral recognition, IFN signals through the type I IFNα/β receptor (IFNAR) leading to the activation of Janus kinase 1 and/or tyrosine kinase 2 (JAK1/TYK2) and recruitment of signal transducer and activator of transcription 1 (STAT1) for phosphorylation and dimerization. The phosphorylated STAT1 dimer then binds IRF9 to form the interferon-stimulated gene factor 3 complex (ISGF3) complex, which binds IFN-stimulated response elements located in ISGs, resulting in the activation of antiviral genes. Some of the most well-studied antiviral genes include *OAS1* and *MxA*. OAS1 is dsRNA-dependent synthetase that activates the endoribonuclease RNase L to degrade ssRNA while MxA proteins bind target viral proteins to inhibit viral replication. The expression of these ISGs induces an antiviral state. Viruses have developed mechanisms to antagonize the type I IFN response. The ability of E1A protein to decrease IRF9 levels, thereby inhibiting the type I IFN pathway, is shown. Viral E1A protein promotes cyclin E expression, which in turn, binds to and promotes the activation of CDK2. Activated CDK2 promotes viral replication. Palbociclib increases IRF9 expression and promotes cyclin E/CDK2 activation. Thus, the ability of the oncolytic virus to replicate and cause cytotoxicity will be dependent on the level of activation of type I IFN and cyclin E/CDK2.

Our studies clearly show that acquired resistance to palbociclib results in a decrease in type I IFN activation, an increase in CAR levels, and the constitutive activation of CDK2 in ER+ MCF7 cells. These underlying mechanisms of palbociclib resistance provide the molecular basis for their increased permissiveness to OAdmCherry. A model summarizing our findings in ER+ MCF7 breast cancer cells is shown in Figure 8. Given that IRF9 is a part of the ISGF3 complex, the decrease in IRF9 and downstream antiviral targets, such as MxA and OAS1, observed in MCF7/pR cells readily explains the increased ability of OAdmCherry to replicate in these cells. While viruses have evolved mechanisms to evade the IFN-induced antiviral responses of IFN-competent hosts, their ability to replicate is greatly enhanced in IFN-defective hosts [27]. Our IRF9 siRNA data in MCF7/pS cells support the role of IRF9 in limiting OAdmCherry replication. These findings agree with a prior study showing that increased type I IFN signaling, and in particular upregulation of MxA, is implicated in resistance to the Ad5/3-delta24 OV [28]. While palbociclib treatment resulted in an increase in the antiviral type I IFN signaling in MCF7/pS cells, as shown by the increase in IRF9 and MxA, paradoxically it potentiated OAdmCherry replication and oncolytic activity. We speculate that this was due to the OAdmCherry-driven increase in cyclin E and subsequent CDK2 activation observed in these cells, which was able to override OAdmCherry inhibition driven by the activation of the type I IFN response. We confirmed that cyclin E expression was required for OAdmCherry replication and cytotoxicity in MCF7/pS cells by performing cyclin E siRNA experiments. We demonstrate that cyclin E is required for the survival of MCF7/pR cells but not for OAdmCherry replication and oncolytic activity. Thus, we concluded that the constitutive activation of CDK2 was sufficient to drive virus replication in these cells. Previous work demonstrating that the cyclin E-driven activation of CDK2 is required for OAdmCherry activation supports this claim [31].

We observed that OAdmCherry demonstrated significant oncolytic activity against ER− palbociclib-sensitive MDA-MB-231 breast cancer cells; however, the addition of palbociclib blocked viral replication and cytotoxicity. Interestingly, this inhibitory effect of palbociclib on viral replication and activity was independent of IRF9 expression. Future studies are needed to determine the mechanism by which palbociclib inhibits OAdmCherry replication and oncolytic activity. Notably, ER− palbociclib-resistant MDA-MB-231 cells displayed low levels of CAR and elevated type I IFN response compared to the ER− palbociclib-sensitive MDA-MB-231 cells, which rendered these cells resistant to the effects of OAdmCherry.

An important limitation of the work presented here is that the models used in this study were generated by chronically exposing the cells to palbociclib, and thus, it is possible that they do not fully recapitulate the clinical setting due to changes that could have been introduced in culture over time. However, our results are in line with a wealth of preclinical and clinical studies demonstrating that cyclin E overexpression and IFN deregulation are often observed after acquired resistance to CDK4/6 inhibition [32,34,52,53,54,55]. It will be important to determine whether the differences in the mechanisms of resistance to CDK4/6 inhibition observed in our studies of ER+ and ER− breast cancer cell models are also observed in patients receiving these regimens.

The differences in permissiveness to OAdmCherry observed in the cell lines tested have two implications. First, ER+ and ER− breast cancer cells with acquired resistance to palbociclib have distinct phenotypes driven by their different underlying molecular mechanisms of resistance. These phenotypes are key determinants of whether or not they will respond favorably to oncolytic viruses. Second, ER expression status might determine the response of breast cancer cells to CDK4/6 inhibitors. The role of ER in the development and progression of breast cancer has been extensively described. Of particular interest for our studies, the activation of ER and progesterone receptor (PR) has been shown to inhibit OAS1 and type I IFN signaling, respectively, in breast cancer models [56,57,58]. We speculate that this regulation of type I IFN signaling by ER may explain why ER+ and ER− palbociclib-resistant breast cancer cells display an opposite pattern of type I IFN signaling. Our results also raise the question of whether ER signaling regulates CAR expression. Future studies are needed to address these questions.

In summary, we have uncovered that OAdmCherry exploits various mechanisms of resistance to palbociclib to increase replication and oncolytic activity in ER+ MCF7 cells. These mechanisms of resistance include decreased type I IFN status, increased CAR expression, and the constitutive activation of CDK2. Conversely, OAdmCherry was unable to replicate in ER− MDA-MB-231 cells with acquired resistance to palbociclib likely due to their low levels of viral entry receptor and increased antiviral state.

## 4. Materials and Methods

### 4.1. Reagents and Antibodies

Palbociclib (PD-0332991) was purchased from Selleckchem (Houston, TX, USA). Phospho-Rb (Ser780) (no. 9307), Rb (D20, no.9313), phospho-STAT1 (Tyr701) (58D6, no. 9167), STAT1 (D1K9Y, no. 14994), IRF9 (D8G7H, no. 28492), OAS1 (D1W3A, no.14498), MxA (D3W7I, no. 37849), phospho-CDK2 (Thr160) (no. 2561), and CDK2 (78B2, no. 2546) antibodies were purchased from Cell Signaling (Beverly, MA, USA). E1A (sc-58658), cyclin E (sc-247), and ERα (sc-8002) antibodies were purchased from Santa Cruz Biotechnology (Dallas, TX, USA). CAR (ab180761) antibody was purchased from Abcam (Cambridge, MA, USA). Beta-actin (A2228) antibody was purchased from Sigma-Aldrich (St. Louis, MO, USA).

### 4.2. Cell Culture, Generation of Palbociclib-Resistant Cells and Palbociclib Treatment

MCF7 (HTB-22) and MDA-MB-231 (HTB-26) cells were purchased from the American Type Culture Collection (ATCC) (Manassas, VA, USA) and maintained at 37 °C with 5% CO_2_. MCF7 and MDA-MB-231 cells were cultured in IMEM (Corning) supplemented with 10% fetal bovine serum (FBS, Invitrogen, Waltham, MA, USA). Drug-resistant cells were established by culturing in media containing palbociclib (0.1–4 μM). Drugs were replenished every 3 days. Cells were subcultured every 1–2 weeks with 25% increments in drug concentration. The resistant cells were established after 6 months and maintained in the presence of 1 μM palbociclib. For experiments, palbociclib-sensitive cells were treated with either the vehicle (H_2_O) (Ctrl) or 500 nM palbociclib. Palbociclib-resistant cells were treated with either 1 μM (Ctrl) or 1.5 μM palbociclib (palbociclib, Selleckchem, Houston, TX, USA). Cells were authenticated by the short tandem repeat (STR) assay (Genetica DNA Laboratories, Cincinnati, OH, USA).

### 4.3. Cell Proliferation Assays

Cells were seeded in triplicates at a density of 5000 cells/well in 96-well plates 24 h prior to the addition of treatment. Cell numbers were calculated using the FluoReporter dsDNA quantitation kit (Molecular Probes) at the time of treatment (0 h) and 72 h after treatment following the manufacturer’s instructions. Cell proliferation was calculated as a function of the number of cells compared to the control at time zero according to the formula: % cell proliferation = [(cells_72 h_ − cells_0 h_)_drug_/(cells_72 h_ − cells_0 h_)_vehicle_] × 100%. The half-maximal inhibitory concentration (IC_50_) of palbociclib was calculated using a sigmoidal regression model using Graphpad Prism, version 8.0 (GraphPad, San Diego, CA, USA).

### 4.4. Cell Cycle Analysis

Cells were seeded in duplicates at a density of 1 × 10^5^ cells/well in 6-well plates 24 h prior to palbociclib treatment. Cells were collected, centrifuged at 2000 rpm for 5 min, washed with ice-cold PBS, fixed with cold 70% ethanol at −20 °C overnight, and stained with 50 μg/mL propidium iodide (Sigma-Aldrich, St. Louis, MO, USA). FACS analysis was performed using a FACSCalibur (BD Biosciences, San Jose, CA, USA) and analyzed using FlowJo (BD, Franklin Lakes, NJ, USA).

### 4.5. RNA Extraction and Next-Generation Sequencing

Total RNA was extracted using the RNeasy kit (Qiagen, Germantown, MD, USA) following the manufacturer’s instructions. Libraries were prepared using the TruSeq Stranded mRNA LT Sample Prep Kit- Set A (Cat# RS-122-2101) with poly-A enrichment. Sequencing was performed on the University of Louisville Center for Genetics and Molecular Medicine’s (CGeMM, Louisville, KY, USA) Illumina NextSeq 500 using the NextSeq 500/550 1 × 75 cycle High Output Kit v2 (Cat# FC-404-2005). A second run was performed to increase the number of reads. For each run, 72 single-end raw sequencing files (.fastq) representing four conditions with three biological replicates and four lanes per replicate were downloaded from Illumina’s BaseSpace [59] (https://basespace.illumina.com/). Sequences were directly aligned to the Homo sapiens hg38 reference genome assembly (hg38.fa) using tophat2 (version 2.0.13). A *q*-value cutoff ≤ 0.05 with |log_2_FC| was used to determine differential expression. RNAseq data available (GEO accession number GSE130437).

### 4.6. Adenoviral Vectors

A replication-deficient adenoviral vector expressing green fluorescent protein under regulation of a cytomegalovirus (CMV) promoter was used as a negative control for virus replication as previously described [60]. The conditionally replicating adenovirus expressing mCherry red fluorescent protein on the capsid was constructed by homologous recombination in *Escherichia coli* (BJ5183 strain) using a fiber gene–modified AdEasy-1 backbone vector AdEz-F5/3 (Ad5Δ*E1*/Δ*E3*-F5/3) and a modified pShuttle vector pSlΔ24-pIX-mCherry. This shuttle vector contained the mCherry coding sequence inserted downstream of the Ad5 minor capsid pIX gene to generate a C-terminal pIX fusion and a 24-basepair deletion in the Ad5 *E1A* gene coding sequence (delta 24) [61].

### 4.7. Combined Therapies

Cells were plated in 24-well plates at a density of 5 × 10^4^ cells/well and allowed to attach for 24 h. Palbociclib-sentitive cells were treated with either the vehicle (H_2_O) (Ctrl) or 500 nM palbociclib. Palbociclib-resistant cells were treated with either 1 μM (Ctrl) or 1.5 μM palbociclib (palbociclib) for the indicated time. Viral infection was performed immediately after drug treatment at an indicated MOI concentration. OAdmCherry-mediated CPE was evaluated by crystal violet staining. Suspended cells were removed by aspiration; the remaining adherent cells were then fixed with 3.7% formaldehyde for 3 min at room temperature. The excess formaldehyde was washed with PBS; the cells were then stained using 1% crystal violet at room temperature for 3 min. Excess crystal violet was washed away with PBS. Plates were then scanned using an HP Scanjet 4070 scanner (HP). The remaining crystal violet was then solubilized with a 2% sodium dodecyl sulfate (SDS) solution, and the sample absorbances were measured at 590 nm using a Synergy HT Multi-Mode Microplate Reader (Bio-Tek, Winooski, VT, USA). The absorbance (OD) values of each treatment were then normalized to mock-treated cells converting each sample OD into the percent (%) of cell number according to the formula: cell number % = (OD of treated cells/OD of mock-treated cells) × 100% as described previously [62].

### 4.8. Adenovirus Titer Assay

For combined treatment, cells were infected with OAdmCherry alone or in combination with palbociclib as described previously. At the indicated times, supernatants were collected and centrifuged for 10 min at 14,000 rpm. Supernatants were then transferred to a new tube to eliminate cell debris and/or cells in suspension that may contain adenovirus. For siRNA experiments, total virus production was measured by combining both supernatant and cell fractions. The supernatants were transferred to a tube, and cells that remained attached to the bottom of the well were harvested with 200 µL of a non-enzymatic cell dissociation solution (Cellstripper, Corning, Tewksbury, MA, USA). Cells and supernatant fractions from their respective treatment were combined in one tube and subjected to 3 freeze–thaw cycles to release viruses contained within cells prior to titration. Supernatants or total virus production were diluted serially by using median tissue culture infective dose or the amount of a pathogenic agent that will produce pathological change in 50% of cell cultures inoculated (TCID50) or by using the end-point dilution method with HEK-293 cells seeded on 96-well plates. Briefly, HEK 293 cells were seeded in 96-well plates at a density of 1 × 10^3^ cells/well and then infected with 5-fold serially diluted viruses. CPE was recorded and scored after incubation for 7 days. The reduction percentage in virus titer is calculated by the formula: reduction % = [(titer of control group − titer of experimental group)/titer of control group] × 100% [31,63].

### 4.9. Western Blot Analysis

Whole cell lysates were harvested using RIPA buffer (Thermo Fisher Scientific, Waltham, MA, USA) supplemented with protease and phosphatase inhibitors. Protein concentration was determined using the BCA protein assay kit (Thermo Fisher Scientific) following the manufacturer’s instructions. Proteins were separated on 10% or 4–20% Mini PROTEAN TGX gels under reducing conditions and transferred to Immun-Blot PVDF membranes (Bio-Rad, Hercules, CA, USA). The membranes were blocked with 5% BSA or 5% nonfat milk in TBS-T (0.1% Tween20) and immunoblotted with the indicated antibodies. HRP-conjugated goat anti-rabbit (Invitrogen) and anti-mouse IgG (Sigma-Aldrich, St. Louis, MO, USA) were used as secondary antibodies. Amersham ECL Prime Western blotting detection reagent (GE Healthcare, Wauwatosa, WI, USA) was used to detect immunoreactive bands. The bands were visualized on autoradiography film BX (MidSci, Valley Park, MO, USA). Quantitative densitometry was performed with UN-SCAN-IT (Silk Scientific, Orem, UT, USA). Signal density was normalized to the corresponding loading control.

### 4.10. siRNA Transfection

Cells were seeded in 6-well plates at a density of 1 × 10^5^ cells/well in 2 mL of complete medium 24 h prior to transfection. Transfections were performed using Lipofectamine RNAiMAX (Invitrogen) following the manufacturer’s instructions. Cells were allowed to incubate for 5 h prior to infection with either AdGFP or OAdmCherry. Cells were harvested 24 h (Cyclin E experiments) or 48 h (IRF9 experiments) post-infection and analyzed by Western blot, fluorescence microscopy, titer assay and crystal violet. The following siRNAs were used: IRF9 siRNA#1, (s20290, Ambion, Waltham, MA, USA); IRF9 siRNA#2 (s20291, Ambion); Ctrl siRNA for IRF9 (4390843, Ambion), cyclin E siRNA SMART pool (M-003213-02-0005, Dharmacon, Lafayetter, CO, USA), Ctrl siRNA pool for cyclin E (D-001206-14-05, Dharmacon).

### 4.11. Fluorescence Microscopy

Cells were infected with either AdGFP or OAdmCherry and inspected for the expression of reporter genes GFP or mCherry, respectively, at the indicated times. Images were taken at 20× magnification with the EVOS FL Imaging System (Thermo Fisher Scientific). GFP was visualized at 357/44 and 447/60 nanometers (nm) excitation and emission, respectively; mCherry was visualized at 587 and 610 nm excitation and emission, respectively.

### 4.12. Statistical Analysis

Results are reported as the mean ± SEM of at least three independent experiments unless otherwise indicated. Statistical analysis was performed by the two-tailed Student *t*-test (independent variable) using Graphpad Prism, version 8.0 (GraphPad). *p* values < 0.05 were considered to be statistically significant.

## 5. Conclusions

Overall, our study provides the mechanistic base for a novel combination regimen consisting of palbociclib and oncolytic virotherapy for the treatment of ER+ breast cancer, particularly those that are palbociclib-resistant. Our findings suggest that combining palbociclib with an OAd will increase the efficacy of palbociclib and prove effective against palbociclib-resistant ER+ breast cancer.

## Supplementary Materials

The following are available online at https://www.mdpi.com/2072-6694/11/5/684/s1, Figure S1: Validation of IRF9-specific siRNAs.
