# Supplementary material for: Targeting Palbociclib-Resistant Estrogen Receptor-Positive Breast Cancer Cells via Oncolytic Virotherapy
Source: Cancers (Basel). 2019 May 16;11(5):684. doi: 10.3390/cancers11050684 (PMC6563125; doi:10.3390/cancers11050684)
Supplement: Supplementary file 1 [file cancers-11-00684-s001.pdf]

Supplementary Material

Targeting Palbociclib-Resistant Estrogen Receptor-Positive Breast Cancer Cells via Oncolytic Virotherapy

Nadiia Lypova, Lilibeth Lanceta, Alana Gipson, Stephanie Vega, Rodolfo Garza-Morales, Kelly M. McMasters, Jason Chesney, Jorge G. Gomez-Gutierrez and Yoannis Imbert-Fernandez

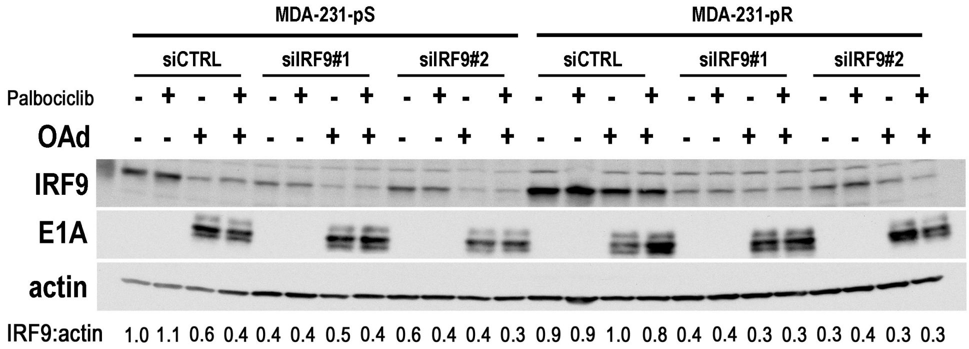

Figure S1. Validation of IRF9-specific siRNAs. Cells were transfected with either control or two IRF9-specific siRNAs followed by AdGFP or OAdmCherry at a multiplicity of infection (MOI) concentration of 5 alone or in combination with 500 nM palbociclib for 48 h. IRF9 and E1A viral protein expression was assessed by immunoblotting. Quantitative densitometry analysis is shown as the ratio of IRF9 to actin protein normalized to MCF7/pS vehicle-treated cells.

© 2019 by the authors. Licensee MDPI, Basel, Switzerland. This article is an open access article distributed under the terms and conditions of the Creative Commons Attribution (CC BY) license (http://creativecommons.org/licenses/by/4.0/).
